# Etoposide-induced DNA damage is increased in p53 mutants: identification of ATR and other genes that influence effects of p53 mutations on Top2-induced cytotoxicity

**DOI:** 10.18632/oncotarget.28195

**Published:** 2022-02-14

**Authors:** Daniel Menendez, Jay R. Anand, Carri C. Murphy, Whitney J. Bell, Jiaqi Fu, Nadia Slepushkina, Eugen Buehler, Scott E. Martin, Madhu Lal-Nag, John L. Nitiss, Michael A. Resnick

**Affiliations:** ^1^Chromosomal Stability Group, Genome Integrity and Structural Biology Laboratory, NIEHS, NIH, Durham, NC 27709, USA; ^2^Department of Pharmaceutical Sciences, College of Pharmacy, University of Illinois, Rockford, IL 61107, USA; ^3^Functional Genomics Laboratory, National Center for Advancing Translational Sciences, NIH, Bethesda, MD 20850, USA; ^4^Environmental Cardiopulmonary Disease Group, Immunity, Inflammation and Disease Laboratory, NIEHS, NIH, Durham, NC 27709, USA; ^5^Department of Pathology and Laboratory Medicine, University of North Carolina at Chapel Hill, Chapel Hill, NC 27599, USA; ^*^These authors contributed equally to this work

**Keywords:** tumor suppressor, siRNA screen, synthetic lethality, ICE assay, Top2 covalent complexes

## Abstract

The functional status of the tumor suppressor p53 is a critical component in determining the sensitivity of cancer cells to many chemotherapeutic agents. DNA topoisomerase II (Top2) plays essential roles in DNA metabolism and is the target of FDA approved chemotherapeutic agents. Topoisomerase targeting drugs convert the enzyme into a DNA damaging agent and p53 influences cellular responses to these agents. We assessed the impact of the loss of p53 function on the formation of DNA damage induced by the Top2 poison etoposide. Using human HCT116 cells, we found resistance to etoposide in cell growth assays upon the functional loss of p53. Nonetheless, cells lacking fully functional p53 were etoposide hypersensitive in clonogenic survival assays. This complex role of p53 led us to directly examine the effects of p53 status on topoisomerase-induced DNA damage. A deficiency in functional p53 resulted in elevated levels of the Top2 covalent complexes (Top2cc) in multiple cell lines. Employing genome-wide siRNA screens, we identified a set of genes for which reduced expression resulted in enhanced synthetic lethality upon etoposide treatment of p53 defective cells. We focused on one hit from this screen, ATR, and showed that decreased expression sensitized the p53-defective cells to etoposide in all assays and generated elevated levels of Top2cc in both p53 proficient and deficient cells. Our findings suggest that a combination of etoposide treatment with functional inactivation of DNA repair in p53 defective cells could be used to enhance the therapeutic efficacy of Top2 targeting agents.

## INTRODUCTION

Topoisomerases are ubiquitous enzymes involved in many aspects of DNA metabolism including DNA replication, transcription and recombination. Type I topoisomerases interconvert topological isomers of DNA by generating transient single strand breaks while type II enzymes use transient DNA double strand breaks (DSBs) to alter DNA structures [1, 2]. Topoisomerases mediate topological changes in DNA through these breaks that involve a protein/DNA covalent intermediate. Although short-lived, various small molecules and other conditions can trap covalent intermediates generating lesions with protein covalently bound to DNA at the DSB site, potentially resulting in genome instability or cytotoxicity. Because these small molecules lead to trapped topoisomerases on DNA, they are referred to as topoisomerase poisons.

Human cells contain two topoisomerase II isozymes encoded by genes *TOP2α* and *TOP2β* [3]. The epipodophyllotoxin etoposide and most other Top2 poisons target both Top2 isoenzymes [4]. Since Top2 poisons generate DNA damage, the cellular response is dependent on DNA repair pathways. For example, defects in DSB repair result in hypersensitivity to Top2 poisons [5, 6]. Proteolytic repair functions [7] and various repair nucleases [8, 9] can remove covalently bound enzyme. DNA damage response (DDR) pathways resulting in downstream events including γH2AX signaling appear to require processing of Top2 covalent complexes (Top2cc) to generate damage signals [10].

The tumor suppressor p53 is a stress-responsive transcription factor that regulates expression of genes important for cell cycle arrest, apoptosis and DNA repair [11]. Stresses such as DNA replication perturbations and DNA damage trigger p53 activation. p53 lies at the intersection of upstream signaling cascades (e.g., ATM/ATR kinase pathways) and downstream DDR pathways, including DNA repair pathways such as base excision repair and homologous recombination [12]. The functional status of the tumor suppressor protein p53 markedly impacts the sensitivity of cancer cells to a variety of chemotherapeutic agents by diverse mechanisms, and tumor cell chemotherapy resistance is often attributed to p53 regulation of apoptotic pathways. As a central player in cancer biology over 50% of human cancers carry *TP53* mutant defects [13]. The multiple roles for p53 present opportunities to specifically target p53 defective cells, provided that wild type p53 responses include regulation of functions that enhance cell survival. Because toxicity of chemotherapeutic agents may limit doses, strategies for selectively sensitizing p53-deficient cancer cells to anticancer drugs are predicted to have a potential clinical impact.

We hypothesized that there exist a group of genes that would result in increased sensitivity of p53 mutants to chemotherapeutic agents when their expression was reduced. This feature could prove clinically useful for addressing human p53-defective tumors. We describe a unique vulnerability of p53 deficient cell lines to agents targeting Top2. Using well-defined quantitative assays, we found that exposure to etoposide leads to a greater accumulation of topoisomerase/DNA complexes in p53-defective cells compared to p53-proficient cells, suggesting that p53 regulates genes that are important for repairing of DNA damage arising from Top2. We carried out a genome-wide RNAi screen for genes whose reduction in expression specifically increased etoposide sensitivity of p53-deficient cells. We term the genes identified in this assay *Synthetic Enhanced Lethal* (SEL) genes. Based on the genes identified in the screen, we observed that ATR inhibition strongly sensitized p53 deficient cells to etoposide. Since ATR inhibition also increased etoposide-induced topoisomerase/DNA complexes, we suggest that this screen can be employed to identify genes that are important for repairing Top2-induced damage in p53-defective cancer cells and can identify genes that can be targeted to enhance etoposide efficacy.

## RESULTS

### Levels of Top2-mediated DNA damage due to acute etoposide exposure are increased in p53 defective cells

Our long-term goal has been to identify contexts where loss of p53 function could be exploited for therapeutic benefit. This led us to examine roles of p53 in response to common anticancer agents. Our model system is based on the human colon HCT116 cancer cell line with defined p53 alterations [14]. As previously shown [15], p53 levels in HCT116 wild-type (WT) p53 increased with etoposide treatment in a concentration-dependent manner (Supplementary Figure 1). In HCT116 *p53*^−/−^ cells stably expressing mutant p53^R273H^, a commonly occurring cancer mutation [16], etoposide treatment induced accumulation of p53 protein, indicating that this mutant p53 can be stabilized as well as its WT counterpart after exposure to DNA damaging agents.

We examined the consequences of p53 loss-of-function or the dominant-negative p53^R273H^ mutation on levels of Top2cc induced by etoposide. Previously, we found that downregulation of DNA repair functions such as Rnf4 leads to elevated levels of Top2cc in the presence of etoposide [17], indicating that DNA repair defects can lead to enhanced levels of protein/DNA covalent complexes. To assess the possible role of p53 in the repair of topoisomerase-induced damage, we examined levels of Top2cc induced by etoposide in the absence or presence of normal p53 function. A standard assay for measuring Top2cc is the ICE (*In vivo* Complex of Enzyme) assay [18, 19]. The assay uses rigorous DNA purification by cesium chloride centrifugation to eliminate proteins associated with DNA by non-covalent interactions. Proteins covalently bound to DNA co-purify with DNA and can be quantified by antibody detection. To make quantitative comparisons, we incorporated two modifications. First, we used purified topoisomerases to generate a standard curve that allows determination of the mass of topoisomerases covalently trapped on DNA in the presence of etoposide. Second, we normalized DNA loading on nitrocellulose membranes using anti-DNA antibodies. These two measures enabled us to calculate the number of Top2cc per cell under various experimental conditions (detailed in Material and Methods section).

**Figure 1:**
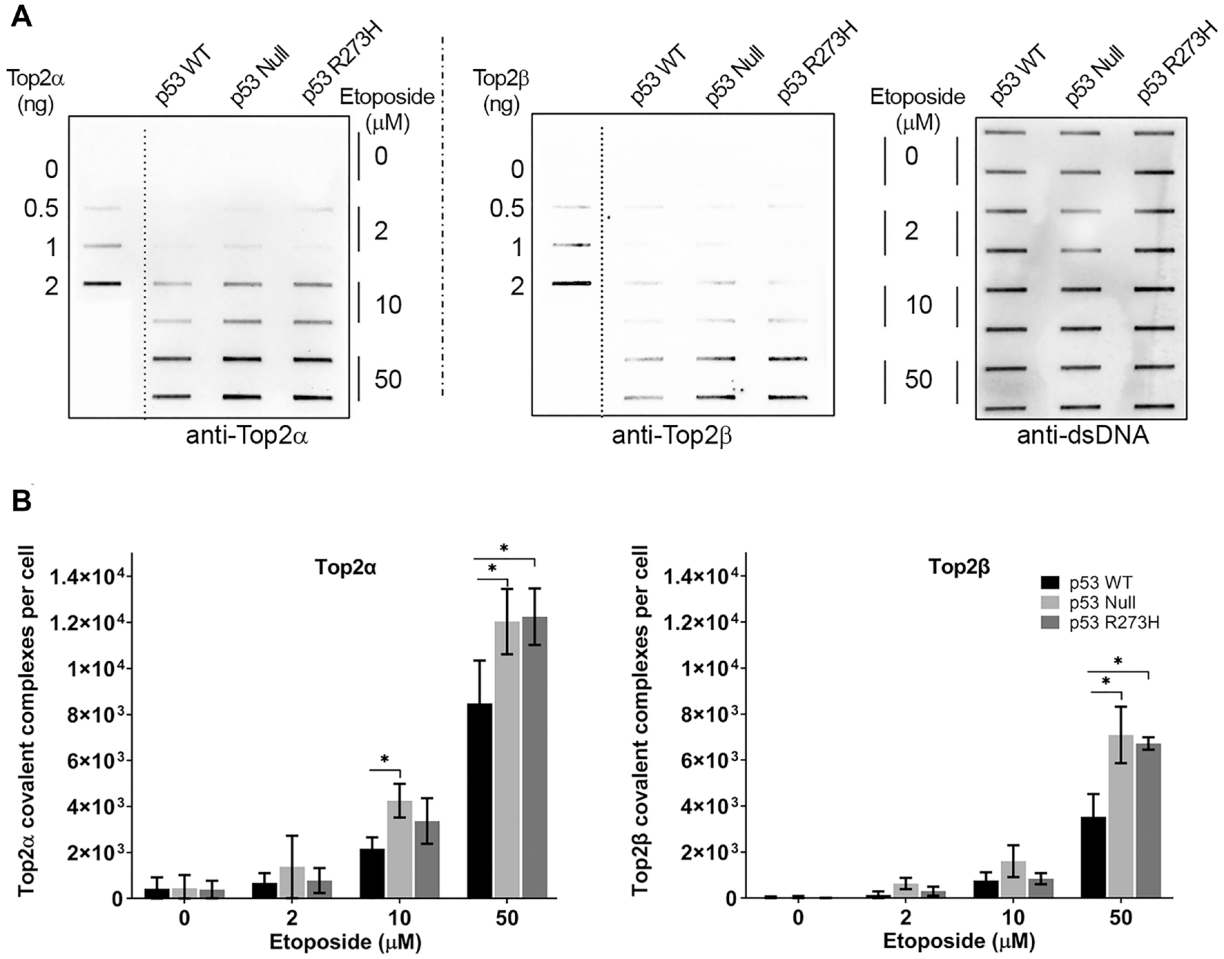
p53 functional status influences etoposide - induced Top2-DNA complexes. (**A**) The ICE assay was performed in isogenic HCT116 cell lines following etoposide treatment for 1 hr at the doses indicated; representative immunoblots for Top2α, Top2β and dsDNA are shown. (**B**) Quantification of Top2cc per cell. *P* values <0.05 were considered significant (^*^).

To establish how the functional status of p53 influences the Top2cc formation in response to etoposide, we applied the modified ICE assay to *p53*^+/+^, *p53*^−/−^ (hereafter, referred to as null), and p53^R273H^ expressing HCT116 cells. Figure 1A shows results of a typical ICE assay of samples for HCT116 cells treated with etoposide for one hour, along with the purified Top2α and Top2β aliquots and a demonstration of consistent DNA loading. Cells treated with etoposide show a dose-dependent increase in both Top2α covalent complexes (Top2αcc) and Top2β covalent complexes (Top2βcc) (Figure 1B). There was no significant difference in Top2cc levels at 2 μM etoposide between the three cell lines tested. However, at 10 μM and 50 μM etoposide, there was a significant increase in Top2αcc and Top2βcc levels in p53 null compared to *p53*^+/+^ cells. The cells expressing the p53^R273H^ also showed a significant difference of Top2αcc and Top2βcc, but only in cells treated with 50 μM etoposide. Overall, these results show that functional p53 status in HCT116 cells affects levels of Top2cc.

To validate the generality of this observation, we used human lung carcinoma A549 cell that express WT p53 and isogenic cells with reduced levels of p53. The reduction was accomplished by a lentiviral vector harboring short hairpin RNAs that target TP53 mRNA, resulting in nearly undetectable levels of p53 protein after etoposide treatment [20] (Supplementary Figure 2A). Interestingly, while HCT116 cells showed about twice the amount of Top2αcc as Top2βcc, A549 cells have approximately equivalent numbers of Top2αcc and Top2βcc at equivalent etoposide doses (Supplementary Figure 2B and 2C). Nonetheless, as seen in HCT116 cells, *p53*^+/+^ cells have lower levels of Top2αcc and Top2βcc than p53 null cells. Taken together, these experiments show that the loss of p53 results in an increase in Top2cc level, suggesting a deficiency in repair of Top2 induced DNA damage.

### Identification of reduced ATR as an SEL gene for etoposide sensitivity using RNAi reduced-expression screening

**Figure 2:**
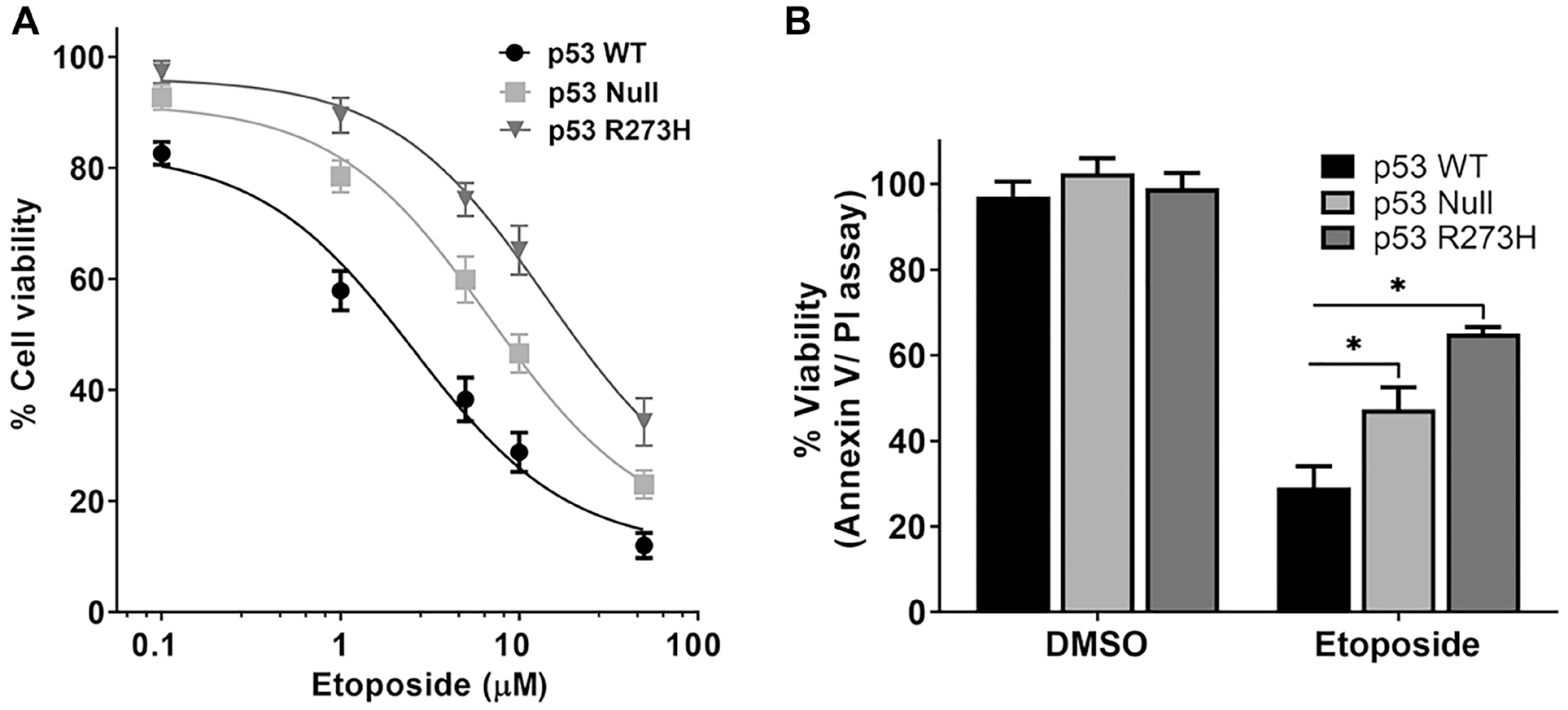
TP53 genotype-dependent cytotoxic effect of etoposide in HCT116 colon cancer cells. Cell viability and apoptosis were evaluated by the (**A**), MTS assay and by the (**B**) Annexin V-FITC/ PI assay, respectively, after 72 hr of incubation with etoposide at the doses (μM) indicated. Data are presented as mean ± SDs of three separate experiments, carried out in triplicate. *P* values < 0.001 were considered significant (^*^) when compared to p53 WT cells.

Similar to a previous report [21], we found in an MTS cytotoxicity assay that *p53*^+/+^ cells were more sensitive to etoposide treatment than p53 null and p53^R273H^ cells. A modest difference in sensitivity was observed at 24 hr and 48 hr post-treatment. However, there was a substantial difference between cell lines at 72 hr (Figure 2A and Supplementary Figure 3). Apoptosis was also assessed at 72 hr following 10 μM etoposide exposure using Annexin V/PI staining. Cells expressing p53^R273H^ were more resistant to etoposide than the p53 null or p53 WT cells (Figure 2B). Our findings indicate that p53-proficient cells have reduced levels of Top2ccs induced by etoposide compared to cells deficient in p53.

**Figure 3:**
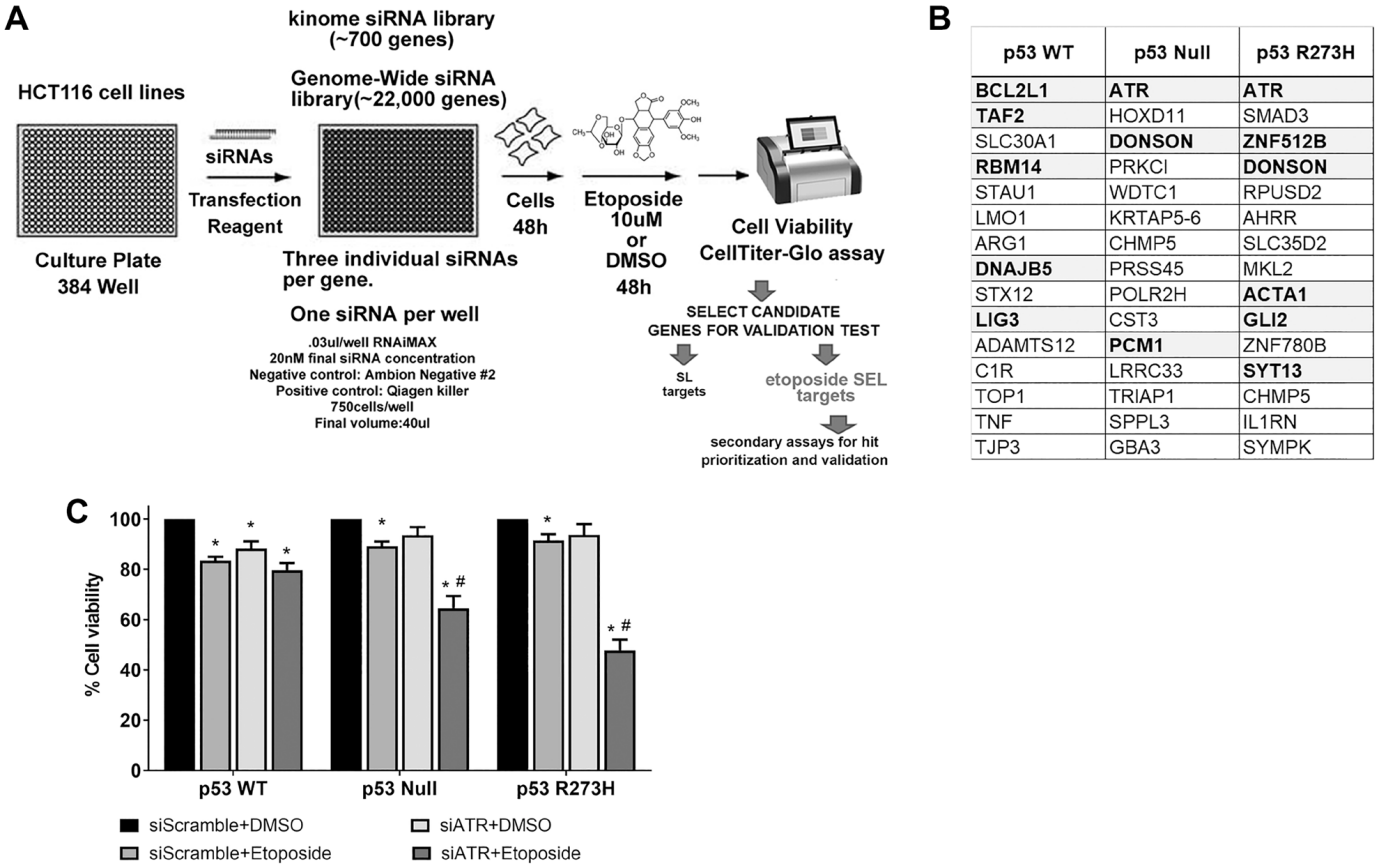
Identification of ATR as an etoposide SEL target in p53 nonfunctional cellular background. (**A**) Schematic representation of the comprehensive screening for etoposide SEL genes. (**B**) Top15 etoposide SEL potential targets from first screen for each HCT116 cell line with different p53 genotype. Validated SEL candidates during the follow-up screens are shown in bold. (**C**) Cell viability measured by MTS assay in HCT116 cell lines with different functional p53 genotypes. Cells were transfected with a pool of siRNA against ATR. Ambion Silencer Select Negative Control #2 was used as internal scramble control. After 48 hr etoposide (10 μM) was added to the cultures, cells were harvested 48 hr later. Data are presented as mean ± SDs from three separate experiments, carried out in triplicate. *P* values < 0.001 were considered significant when compared to their respective DMSO treated cells (^*^) or to its p53 WT siATR+Etoposide condition (^#^).

If p53 regulates the removal of Top2ccs, then transient reduced repair functions might preferentially enhance the sensitivity of human p53 defective cells to etoposide. We conducted siRNA genome-wide screens in HCT116 isogenic WT, null, and p53^R273H^ expressing cells (Figure 3A). The screen was performed using an Ambion Human Genome Silencer Select siRNA library that consists of three different siRNAs targeting 21,584 human genes [22]. For the primary screen, etoposide was added 48 hr post-transfection (10 μM) and luminescence based -viability assay (CellTiter Glo) was assessed 48 hr later. After normalization of each siRNA to the median negative control value of the luminescence intensity, the median absolute deviation (MAD) based z-score was calculated for each siRNA. To select candidates whose knockdown enhanced etoposide sensitivity, the log2 ratio of vehicle-treated cell viability (% siNeg) to etoposide -treated cell viability (% siNeg) was calculated for each siRNA (see Material and Methods). In all cases, genes were considered as potential SEL candidates if at least two of the three siRNAs resulted in MAD values >1.9 and log(p) values greater than -2. From our initial screen, the top 15 targets from each p53 background condition (Figure 3B) were considered for further validation. The complete dataset for the primary screen is described in Supplementary Dataset 1. Pathway analysis of the SEL candidates indicated that these genes are involved in DDR and DNA replication (*ATR, LIG3, TOP1, RBM14, DONSON, POL2RH*), cell cycle regulation (*PCM1, TRIAP1, RBM14, SMAD3, ATR, CHMP5, DONSON*), caspase and apoptosis signaling (*BCL2L1, TRIAP1, TNF, ACTA1*), immune responses (*TNF, SMAD3, IL1RN, PRKCI*) and transcriptional regulation (*TAF2, POLR2H, ARG1, GLI2, ZNF512B, ZNF780B*) among other processes. A further analysis by STRING [23], also identified enrichment for known protein-protein interactions for some of the SEL candidates of each cell line (Supplementary Figure 4).

To further investigate the top 45 candidate SEL genes, we performed two additional rounds of validation using three additional siRNAs of each candidate gene that did not overlap with the siRNAs used in the primary screen. First, we determined the etoposide sensitivity 48 hr post-siRNA transfection in the HCT116 cell. In the second validation, the etoposide treatment after transfection was extended to 72 hr. Based on the combined primary and secondary follow-up screens and applying the criteria used in the primary screen (described above), we identified 12 validated SEL candidates (highlighted in bold, Figure 3B). Representative examples of validated SEL candidates with etoposide sensitivity vs. p53 genotype are shown in Supplementary Figure 5A. The complete dataset and the siRNA sequences used for the follow-up screens is available in Supplementary Dataset 2.

Among the 12 validated candidates in our siRNA screen, the most potent and robust SEL target was the ATR gene (Ataxia-telangiectasia and the Rad3-related kinase). We focused on ATR because of its general impact on S-phase checkpoint regulation and replication-associated damage. Interestingly, our siRNA screens identify ATR as a specific etoposide related SEL target for the null and p53^R273H^ expressing cells (Figure 3C and Supplementary Figure 5A) but did not affect WT p53 cells under our conditions. We confirmed by qPCR that a pool of the best three siRNAs against ATR resulted in a significant knockdown in gene expression (Supplementary Figure 5B).

**Figure 4:**
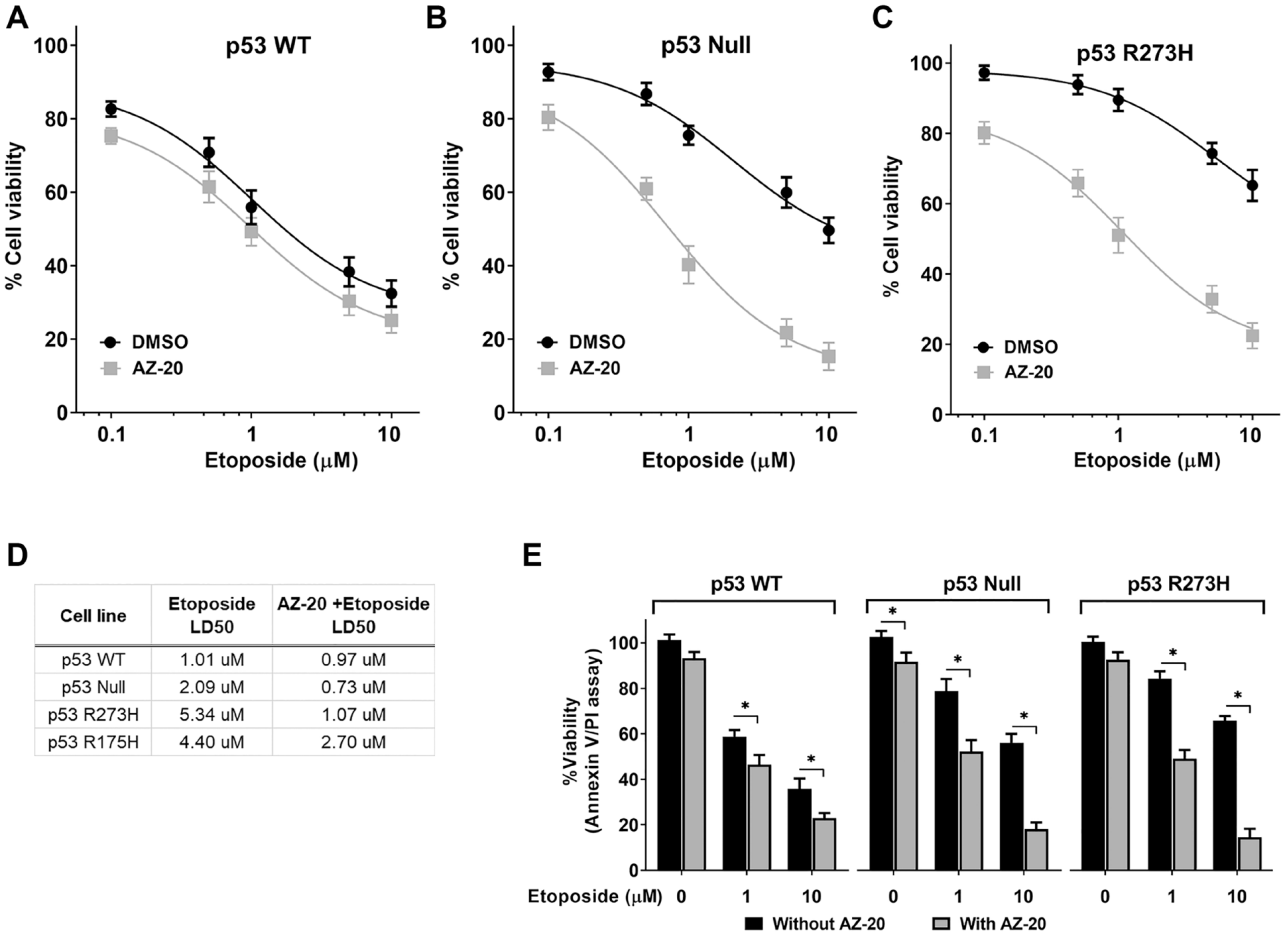
Pharmacological inhibition of ATR increased etoposide sensitivity in p53 nonfunctional cells. Cell viability curves assessed by MTS assay using isogenic HCT116 cell lines for p53 (**A**) WT, (**B**) null or (**C**) R273H mutant following pretreatment with either DMSO or ATR inhibitor AZ-20 (5 nM) for 3 hr, followed by etoposide treatment (10 μM, 72 hr). (**D**) LD50 for etoposide in absence or presence of ATR inhibitor. (**E**) Etoposide sensitivity evaluated by Annexin V/PI assay in the absence or presence of ATR inhibitor. Data are presented as mean ± SDs from three separate experiments, carried out in triplicate. *P* values <0.001 were considered significant (^*^).

Consistent with the results observed in experiments employing siRNA, pretreatment of p53 null and p53^R273H^ HCT116 cell lines with the ATR inhibitor AZ-20 [24] significantly increased sensitivity to etoposide treatment, as shown in Figure 4B and 4C. AZ-20 had a lesser impact on the WT p53 cells (Figure 4A). IC_50_ values for AZ-20 were calculated as the drug concentration necessary to inhibit growth OD590 by 50% compared to the vehicle, control-treated cells (Figure 4D). Both p53 null and p53^R273H^ expressing cells exhibited a significantly higher sensitivity, 3 and 5-fold, respectively, than the p53 WT cells, where AZ-20 does not increase the sensitivity to etoposide. Similarly, using the Annexin-V/ PI assay, we also observed that ATR inhibition results in more than 10-fold increased etoposide sensitivity in p53 null and p53^R273H^ expressing cells (Figure 4E). Interestingly, we observed that for expression of a different hotspot tumor-associated p53 mutant R175H in HCT116 *p53*^−/−^ cells (Supplementary Figure 6A) the ATR inhibition had a modest effect in increasing the etoposide sensitivity, possibly indicating a p53 mutation-specific effect (compare HCT p53^R273H^ in Figure 4C with HCT116 p53^R175H^ in Supplementary Figure 6B). Consistent with this observation, like in HCT116 p53 null and p53^R273H^, we also found a 5- to 10-fold enhanced etoposide sensitivity when ATR is pharmacologically inhibited in other cancer cell lines deficient in p53 (SaOS2 and H1299) or endogenously expressing the p53^R273H^ mutant (MDA-MB-468) (Supplementary Figure 6C). On the other hand, there was no such SEL effect in cancer cell lines expressing other p53 mutations including the partially functional P153A (HCT15) or the conformational mutants R175H (SkBr3) and R280K (MDA-MB-231), suggesting again a potential p53 mutant-specific effect. Overall, we conclude that ATR Inhibition can enhance the etoposide sensitivity in p53 dysfunctional cancer cell lines, potentially in a p53 mutation-specific manner.

### ATR contributes to the repair of Top2 damage

**Figure 5:**
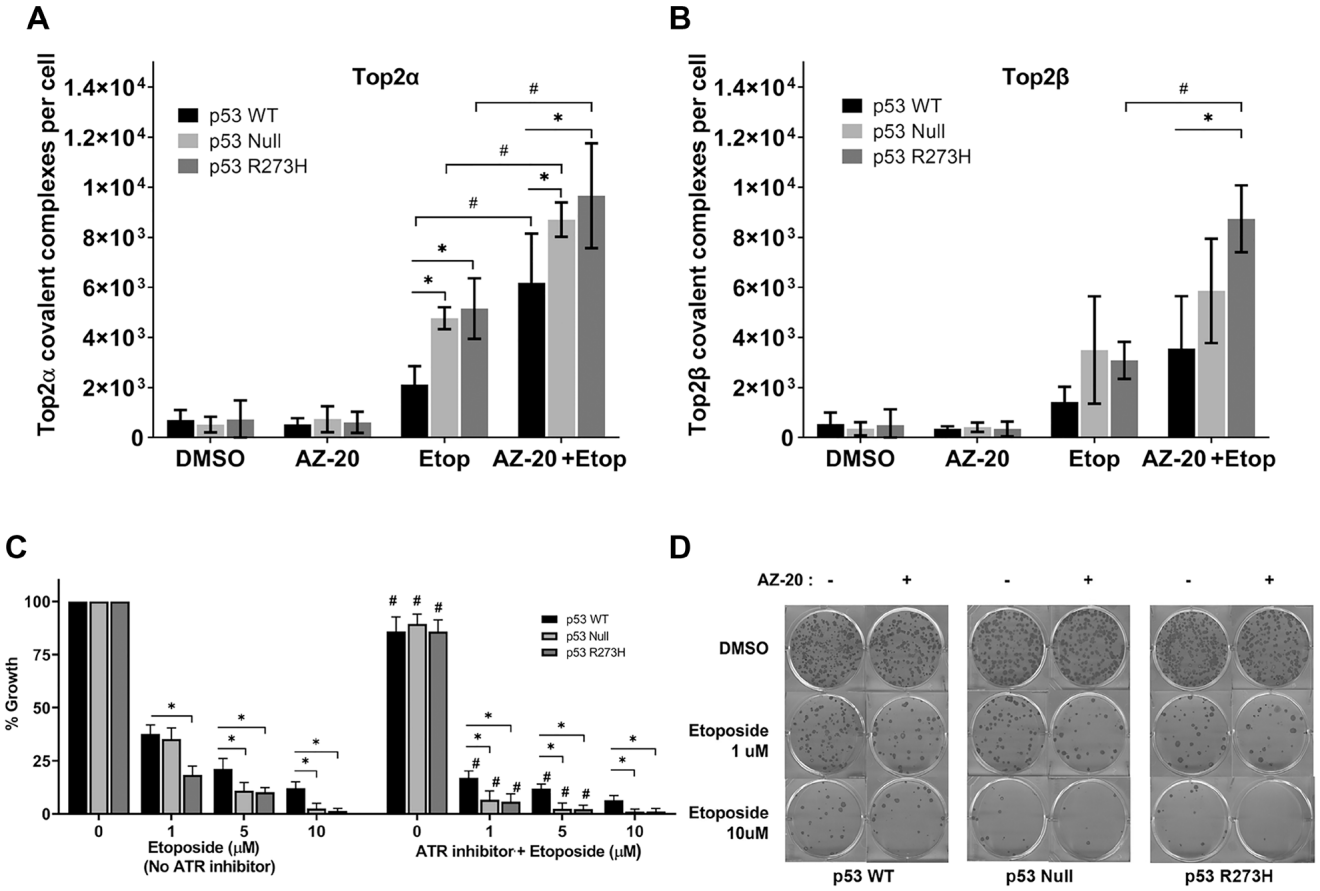
ATR inhibitor AZ-20 generates elevated levels of Top2cc in isogenic HCT116 p53 WT, Null and R273H cell lines. The ICE assay was performed following pretreatment with either DMSO or ATR inhibitor AZ-20 (200 μM) for 0.5 hr, followed by etoposide treatment (10 μM, 2 hr). ICE samples were isolated and levels of trapped (**A**) Top2α and (**B**) Top2β were determined. *P* values < 0.05 were considered significant (^*^,^#^). (**C**) Clonogenic survival after ATR inhibition and etoposide treatment. Cells were treated with DMSO or AZ-20 (5 nM, 2 hr) followed by etoposide treatment (10 μM, 24 hr).The total number of colonies per dish was normalized to vehicle treated (DMSO) controls. *P* values < 0.001 were considered significant when compared to p53 WT cells (^*^) or when compared to their respective etoposide treated cells without AZ-20 (^#^). (**D**) Representative plates for clonogenic experiment. Data are presented as mean ± SDs from three separate experiments.

Based on the sensitivity of p53 deficient cells to ATR inhibition, we examined levels of Top2αcc and Top2βcc in cells treated with AZ-20 and etoposide. Cells were pretreated with 200 μM AZ-20 for 30 minutes before the etoposide (or control solvent) treatment. Cells treated only with 200 μM AZ-20 did not show elevated levels of Top2αcc or Top2βcc (Figure 5A and 5B). As we found previously, p53 deficient HCT116 cells have elevated levels of Top2αcc and Top2βcc compared to *p53*^+/+^ cells. When *p53*^+/+^ cells were treated with 10 μM etoposide plus 200 μM AZ-20, significant increases in Top2αcc and Top2βcc were observed compared to cells treated with etoposide alone (Figure 5A and 5B). The same treatment of p53 null and p53^R273H^ cells also increased the levels of Top2αcc and Top2βcc when compared to cells treated only with etoposide. Thus, inhibition of ATR activities decreases repair of Top2 complexes, independent of p53 status. In terms of repair of complexes, the effect of reducing the functionality of ATR on the repair of Top2 induced damage by AZ-20 appears to be at least additive with the impact of a p53 defect. We also examined the effect of AZ-20 in A549 cells proficient or deficient in p53. As observed for HCT116 cells, pretreatment with AZ-20 alone did not lead to elevated Top2αcc or Top2βcc, whereas AZ-20 plus etoposide led to higher levels of Top2αcc and Top2βcc (Supplementary Figure 7) in A549 cell lines. These results provide additional support for the hypothesis that ATR contributes to the repair of Top2 damage as suggested previously [25].

To further explore how inhibition of ATR impacts the Top2 complexes and selectively sensitizes p53-deficient cancer cells to etoposide, we used a clonogenic assay to evaluate the ability of cells to survive different etoposide concentrations in the presence or absence of the ATR inhibitor AZ-20. In contrast to the metabolic-based viability assay (Figure 2B), which reflects the acute effects of etoposide, the chronic clonogenic cytotoxicity assays showed that p53 WT cells had higher survival than p53-deficient cells at 5 or 10 μM etoposide (discussed below; Figure 5C).The inhibition of ATR in the absence of etoposide had a modest impact on the colony formation for all the cell lines. As expected, the transient inhibition of ATR expression caused a reduction in clonogenic survival in all cell lines examined since the protein ATR is required for efficient DNA replication (Figure 5C and 5D). These results are consistent with the higher accumulation of Top2cc when ATR is inhibited in the presence of etoposide in p53 nonfunctional vs. WT cells.

## DISCUSSION

Eukaryotic Top2 is critical for genome integrity, but its enzymatic activity has the potential to compromise genome integrity and cell survival when progression through its catalytic cycle is compromised [3, 26, 27]. The activity of anticancer drugs that act through topoisomerase-induced DNA damage are an example where progression through the enzyme catalytic cycle is blocked leading to DNA damage. The consequences of this damage can be modified by an extensive repertoire of repair pathways [4, 28]. Trapped topoisomerase covalent complexes are a type of DNA damage that, when combined with DDR defects, provide targeted therapeutic possibilities.

We used purified Top2α and Top2β proteins as standards in the ICE assay to quantitatively assess numbers of covalent complexes induced per cell by etoposide. This enhancement to the ICE assay provides a more detailed picture of the DNA damage induced in the presence of etoposide. Interestingly, HCT116 cells had a 2:1 ratio of Top2αcc to Top2βcc (Figure 1). Assuming that increased levels of Top2αcc and Top2βcc have similar phenotypic consequences, our results suggest that specific targeting of Top2α may not be beneficial in all contexts (i.e., in cells with relatively high levels of Top2βcc) as has been recently suggested [29, 30]. The Top2βcc if present at high levels would be expected to contribute to etoposide cytotoxicity.

We found that in response to the Top2 poison etoposide, loss of p53 leads to elevated levels of Top2ccs (Figure 1, Supplementary Figure 2). We hypothesize that elevated levels of Top2cc in the presence of a Top2 targeting agent result from reduced repair, as has been observed with loss of function of other repair functions [17]. Thus, targeting a DDR pathway in combination with etoposide treatment would be effective in cells defective in p53 [31–34].

Previous findings on combined effects of p53 and Top2 targeting have been contradictory. For example, Hochhauser and colleagues [35]. found an increase in DNA breaks induced by etoposide when p53 was introduced into p53 null cells. These alterations were not due to changes in Top2α or Top2β expression. Similarly, p53 status does not affect the protein expression levels of Top2β [36]. By contrast, Wang et al. [37] found that p53 inhibits expression of Top2α, which might influence Top2-induced DNA damage. Since both Top2αcc and Top2βcc levels are increased in p53 deficient cells we suggest that the most likely explanation is p53 regulation of genes required for repairing Top2-induced DNA damage. Possible targets include a variety of DNA repair entities, such as the proteasome, which has been implicated in the repair of Top2 induced damage and could be affected by p53 status [10]. Also, mutant p53 might lead to enhanced expression of the Top2 specific repair proteins such as Tdp2. However, siRNA mediated knockdown of p53 did not change Tdp2 levels [38], although this needs to be examined in other cellular contexts. The complete set of topoisomerase-specific repair functions remain to be defined, and regulation of these functions by p53 will be an important part of their characterization. Regardless of the detailed mechanism(s), our results provide an explanation in part of the therapeutic index of topoisomerase II targeting anti-cancer drugs.

Functional status of p53 markedly impacts the sensitivity of cancer cells to a variety of chemotherapeutic agents by diverse mechanisms [39]. The impact of changes in various repair steps that p53 may mediate remains to be determined. For example, p53 may regulate the removal of Top2 covalently bound to DNA, subsequent DSB repair and DNA damage signaling steps. While WT p53 cells can be more sensitive to etoposide than p53-defective cells, differences in sensitivities between WT p53 and p53 mutant cells depend upon the assay used. A commonly held view is that etoposide resistance associated with p53 deficiency is due to defects in apoptosis or senescence pathways [40–43], and our results indicate that other processes may be important.

We observed that p53 deficient cells are etoposide-resistant in the short-term MTS determined growth assay but were hypersensitive in clonogenic survival assays, an observation that at first may appear contradictory. The MTS assay provides an acute measure of toxicity across a cell population where cell viability and drug cytotoxicity are assessed by population metabolic activities. However, these assays do not directly measure the reproductive capability of damaged cells. The clonogenic assay evaluates the ability of individual cells to continue to divide and produce multiple cell generations. Even though we observed increased survival following acute exposure to etoposide in p53 nonfunctional cells based on the MTS cell growth assay, in the long term clonogenic survival assay the p53-deficient cells were more sensitive to etoposide than WT p53 cells. The results of the clonogenic assays are consistent with increased levels of unrepaired Top2cc adducts in p53 null and mutant cells (Figure 1 and Supplementary Figure 2).

The etoposide resistance of p53 mutants in the MTS assay motivated us to apply genome-wide screening assays to identify genes whose loss-of-function might ameliorate the etoposide resistance seen int these assays. Based on the results with clonogenic survival assays we expected the gene deficiencies to also specifically reduce the reproductive capacity of p53 deficient cells. Synthetic lethality approaches provide understanding of the function of genes and the interdependence between pathways and signaling networks in tumor cells, as well as targeted anticancer therapy strategies. In our case, we have pursued genes whose temporary decrease in expression significantly reduce the etoposide determined resistance of the p53-defective mutants in the MTS assay, thereby identifying SEL target genes.

**Figure 6:**
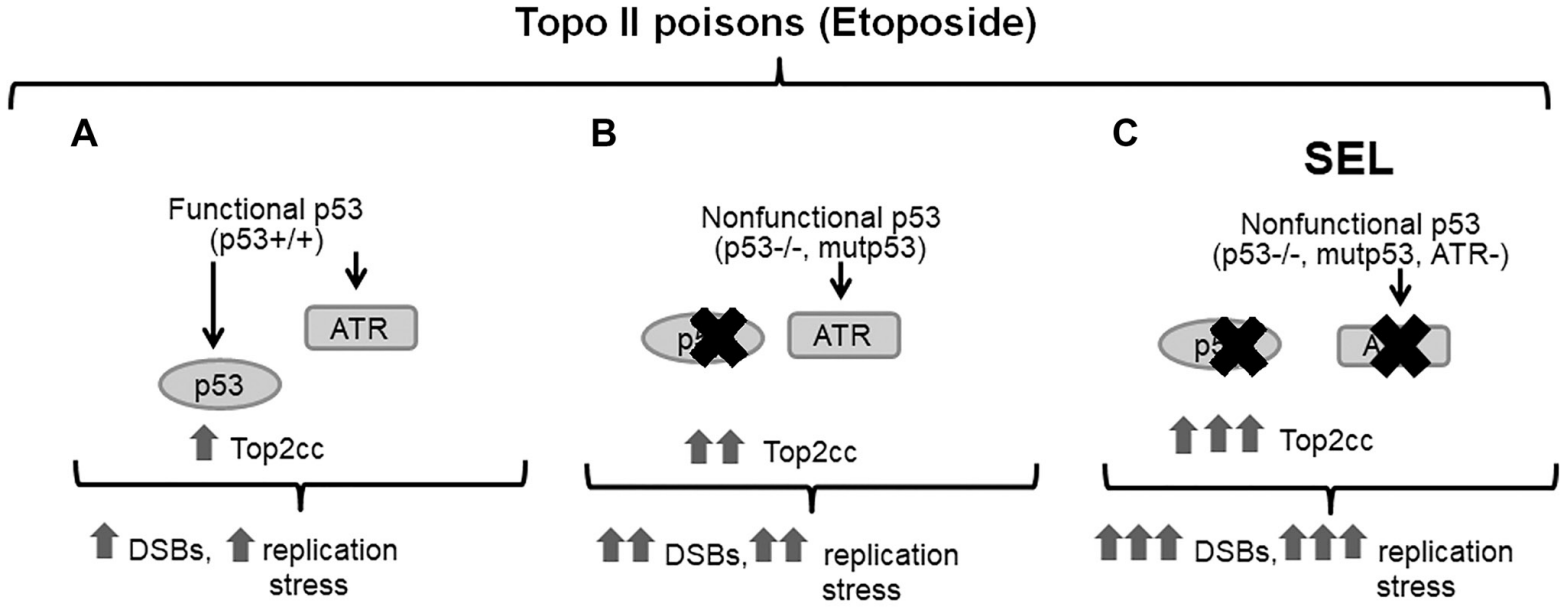
The synthetic lethal interaction between ATR and the p53 pathway in cells exposed to topoisomerase II inhibitor etoposide. Inhibition of ATR leads to accumulation of DSBs from collapsed stalled replication forks or by the replication stress triggered by etoposide inhibition of Top2. In functional WT p53 cells (**A**), with intact cell cycle checkpoints, other compensatory mechanisms reduce proliferation and promote repair. This contrasts with p53-deficient cells (**B**) that have lost G1 checkpoint control and where the initiation of DNA replication continues along with an accumulation of DNA damage. Since p53 deficient cells rely on the ATR/Chk1 pathway S/G2 checkpoints for repairing DNA damage, the ATR inhibition (**C**) results in selective killing of cancer cells due to the accumulation of etoposide-induced Top2cc DNA lesions and higher levels of unrepaired damage. This would lead to increased cell death, possibly due to mitotic catastrophe or as a consequence of loss of G2/M arrest. Normal cells with a functional p53 and G1 checkpoint would be less affected.

In our RNAi screen, the ATR gene was identified as a SEL target for the impact of etoposide on MTS determined growth (Figure 3) of nonfunctional p53 null and p53^R273H^ mutants as well as in colony forming assays (Figure 5C). Reduction in ATR by siRNA or the inhibitor AZ-20, which has anti-colorectal tumor activity *in vivo* at 50 mg/kg per day [24], leads to increased etoposide-trapped Top2cc in both WT and these p53-deficient cells. This is presumably due to a delay and/or deficiency in DNA repair. The more significant impact in the p53 null and p53^R273H^ mutants when compared to p53 WT cells likely relates to increased levels of Top2 induced DNA damage. At cellular level we found that ATR inhibition by AZ-20 resulted in more than 10-fold increased etoposide sensitivity in p53 null and p53R273H expressing cells at doses (10 μM) closely related to the ones reported in serum (5–8 μM) of cancer patients treated orally with etoposide [44]. Our data support the use of ATR inhibition as a therapeutic strategy in combination with etoposide and other agents to target malignancies with cells lacking functional p53, at least for the p53 null and p53^R273H^ defects. The synthetic lethal interaction between ATR and the p53 pathway in cells responding to etoposide is summarized in Figure 6.

Disruption of ATR function impacts cancer cell survival in both the absence and the presence of DNA-damaging agents [45–47]. Since DDR can reduce the effectiveness of chemotherapy agents by activating checkpoints and triggering DNA repair pathways that result in increased tumor cell drug resistance and survival, it is not surprising that ATR is a therapeutic target in cancer cells. Here, we establish that ATR directly impacts levels of etoposide induced Top2 complexes, most likely by affecting the repair of the complexes. Several reports suggest that functional inhibition of ATR can intensify the levels of replication stress in oncogene-driven tumors to boost damage and promote cell death [48–50]. Our data is consistent with previous reports showing that ATR is involved in sustaining the G2 checkpoint after Top2 poisoning [51, 52], opening the possibility that cell death response observed in p53 mutant cells upon downregulation of ATR and etoposide treatment could be a consequence of loss of G2/M arrest.

In addition to ATR, we identified several other potential etoposide SEL targets involved in DDR signaling, including LIG3 and TOP1 for WT p53 cells, both are critical components of DNA repair pathways, and the DNA replication stabilization factor DONSON for nonfunctional p53 mutant cells. Furthermore, enrichment for known protein-protein interactions for some SEL candidates suggests promising avenues for additional studies. Undoubtedly, further studies to validate these and other potential SEL targets in the context of p53 functionality and etoposide treatment will provide more information and clues about targeted anticancer therapy.

Recently, a set of CRISPR-Cas9 inactivated genes were identified that resulted in etoposide hypersensitivity in a p53 deficient environment [53]. As was the case for the screen reported here, Olivieri and colleagues interpreted the hypersensitivity as arising from deficiency in repairing Top2 induced damage. The present study directly addressed the impact of mutant p53 as well as the combined influence of ATR and p53 defect. Given the very different selection scheme employed by Olivieri and colleagues [53] compared to the screen reported here, it is not surprising that there are differences in genes reported as having a significant effect on survival in the presence of etoposide. It is important to note that the CRISPR-Cas9 assay obliterates gene function, whereas the RNAi approach results in a temporary reduction of gene activity. Nonetheless, both screens successfully identified factors known to repair topoisomerase induced damage and represent fertile ground for identifying repair inhibitors to enhance the clinical efficacy of topoisomerase targeting.

Given the wide variation in the transcriptional and biological impact of p53 mutations, such as gain-of-function, we anticipated variations in the effects of SEL genes among p53 mutants. Since many cancer-associated p53 mutations can result in resistance to therapeutic agents, we hypothesize SEL gene targets that would reduce resistance of specific p53 mutants, thereby providing personalized p53 mutant therapies.

Our findings provide new insights for identification of druggable targets. Manipulation of SEL genes could reduce the dose of chemotherapeutic agents necessary for targeted killing of cells harboring mutant p53 as compared to cells with WT p53, thereby enhancing therapeutic efficacy. In addition, the results provide opportunities to address crosstalk between the processes of DNA repair and DDR. They expand our understanding of critical regulatory networks and functional gene groupings in a context of p53 status, as well as provide valuable information about specific categories of p53 mutants. Collectively, our approaches can enhance opportunities for individualized cancer treatments where the nature of the p53 cancer-associated mutation might dictate the protocol.

## MATERIALS AND METHODS

### Cell culture and chemical compounds

We used the following panel of cell lines directly obtained from American Type Culture Collection, ATCC: SaOS2 (Cat# HTB-85, RRID:CVCL_0548), A549 (Cat# CCL-185, RRID:CVCL_0023), NCI-H1299 (Cat# CRL-5803, RRID:CVCL_0060), HCT-15 (Cat# CCL-225, RRID:CVCL_029), MDA-MB-231 (Cat# HTB-26, RRID:CVCL_0062), SkBR3 (Cat# HTB-30, RRID:CVCL_0033) and MDA-MB-468 (Cat# HTB-132, RRID:CVCL_0419). Human colon cancer HCT116 *p53*^−/−^ and *p53*^+/+^ cells were a gift from B. Vogelstein (John Hopkins University, Baltimore, MD, USA). A549 cells stably expressing a scramble or p53-directed shRNA cell lines were established as previously described [54]. All these cell lines were grown in a 37°C incubator with 5% CO2 in DMEM, McCoy’s or RPMI media (Thermo Fisher Scientific Cat# 11995065, Cat# 16600082, Cat# 21870076) supplemented with 10% fetal bovine serum (Cat# 26140079), L-glutamine (2 mM, Cat#25030149), penicillin-streptomycin (100 units/ml; 100 μg/ml, Cat#15140148) (all reagents from Thermo Fisher). All cell lines were cultured within less than six passages following resuscitation and regularly tested for mycoplasma using a MycoAlert Mycoplasma Detection Kit (Lonza, Cat# LT07-118). Cells were plated 18–24 hr before being treated with Etoposide (Sigma-Aldrich, Cat# E1383) unless otherwise indicated. DMSO (0.1% volume, Sigma-Aldrich, Cat# D2650) was used as solvent. The ATR inhibitor (AZ-20, Tocris Bioscience, Cat# 5198) was dissolved in DMSO.

### Generation of stable p53 mutant cell lines

pLenti6/V5-p53_R175H (Addgene plasmid # 22936; http://n2t.net/addgene:22936; RRID:Addgene_22936) and pLenti6/V5-p53_R273H (Addgene plasmid Cat# 22945; http://n2t.net/addgene:22945; RRID:Addgene_22945) were a gift from Bernard Futscher [55]. Lentiviruses were produced by the NIEHS Viral Vector Core and packaged in HEK293T/17 cells (ATCC Cat# CRL-11268, RRID:CVCL_1926) as previously described [54]. To generate stable p53^R273H^ and p53^R175H^mutant expressing cell lines, lentivirus particles were incubated with HCT116 *p53*^−/−^ cell s for 48 hr and blasticidin (2 μg/ml, Thermo Fisher, Cat# A1113902) selection was performed for 2 weeks.

### *In vivo* complex of enzyme (ICE) assay

A modified ICE assay [18] was used to measure absolute levels of Top2cc per cell. Etoposide or DMSO (solvent) was added to the final concentrations indicated for individual experiments, and incubated for 1 h, or as indicated. Following drug treatment, cells were lysed using 1.5 ml 1% (w/v) sarkosyl (Sigma-Aldrich, Cat# 61739). DNA was sheared by passing the cell lysates through a 25-5/8G-needle using 1 ml syringe and volume was adjusted to 3 ml with additional sarkosyl. First, 2 ml of cesium chloride (CsCl, Sigma-Aldrich, Cat# 43568) was added to 4.9 ml OptiSeal centrifuge Tube (Beckman coulter, Cat#362185). Then, 3 ml of the lysate was layered over the CsCl solution in the OptiSeal tube. OptiSeal tubes were loaded on an ultracentrifuge (42,000 rpm, 25°C, 20 h). Supernatant containing free protein was discarded. The DNA pellet was washed with 70% ethanol and was resuspended in 500 μl 1X TE buffer, pH 7.5 (10 mM Tris HCl, pH 7.5; 1 mM EDTA, Thermo Fisher, Cat#15568025, Cat#AM9260G). DNA concentration was determined using spectrophotometer and 2 μg DNA per sample was spotted in duplicate on nitrocellulose membranes using slot blot apparatus. Purified human Top2α or Top2β proteins were also loaded for generating standard curve. The membranes were then immunoblotted with antibodies against Top2α (Bethyl Cat# A300-054A, RRID:AB_221392), Top2β (BD Biosciences Cat# 611493, RRID:AB_398953) or dsDNA (Abcam Cat# ab27156, RRID:AB_470907). Signals were detected using SuperSignal West Femto Maximum sensitivity substrate ECL kit (Thermo Fisher, Cat# 34096)and quantified using ImageJ software. Total Top2α and Top2β covalent complexes per sample were calculated using standard curves for purified Top2α and Top2β, respectively. Absolute Top2αcc and Top2βcc per cell were calculated by first normalizing covalent complexes to DNA loaded and later calculating covalent complexes per 7 picograms of DNA (assuming 7 picograms of DNA per diploid cell).

### siRNA screening

For transfections, 20 μL of serum-free media containing Lipofectamine RNAiMax (0.03 μL, Thermo Fisher, Cat# 13778500) was added to wells of 384-well plates (Corning, Cat#3570) containing pre-stamped siRNA (2 μL 400 nM). Lipid and siRNA were allowed to complex for 45 minutes at ambient temperature before addition of 750 HCT116 cells to yield final transfection mixtures containing 20 nM siRNA in RPMI, 10% FBS. Etoposide (10 uM) or vehicle (0.1% DMSO) was added to the entire plate 48 hr after transfection, and viability (CellTiter Glo; Promega, Cat# G7570) was assayed 72 hr later on a PerkinElmer Envision 2104 Multilabel Reader. The whole genome screening was conducted using the Ambion Human Genome Silencer Select siRNA (Thermo Fisher Scientific, Cat# 4397926), which targets nearly 21,584 human genes with approximately 3 siRNAs per gene. Each siRNA is arrayed in an individual well. Ambion Silencer Select Negative Control #2 (Cat#4390847) and Qiagen Allstars Hs Cell Death control (Cat#1027299) were incorporated on all screening plates for normalization and as positive transfection control, respectively. Plates were rejected and rescreened if they exhibited an assay z′-factor of less than 0.4 or other apparent defects. Follow-up dose–response tests were carried under analogous assay conditions. To select candidate genes modulating etoposide activity, the log2 ratio of vehicle-treated cell viability (%siNeg) to etoposide -treated cell viability (%siNeg) was calculated for each siRNA. Redundant siRNA Analysis (RSA; [56]) was performed on the ratios to rank gene candidates in terms of their ability to sensitize each HCT116 cell line to etoposide. For each plate, the median value of the negative control wells was set as 100% and was used to normalize corresponding sample wells. The “viable cell density” were exported as the percentage of the negative control, and the median absolute deviation (MAD)-based z-score was calculated for each sample [57]. To select candidate genes modulating etoposide activity, the log2 ratio of vehicle-treated cell viability (% siNeg) to etoposide -treated cell viability (% siNeg) was calculated for each siRNA. For the primary screen, the genes that were targeted by at least two independent siRNAs (out of three) resulting in enhanced luminescence production with MAD >1.9 and a log(p) values greater than −2 (used as parameter of effectiveness), were then subjected to follow-up validation screens using three additional independent siRNAs (QIAGEN) with different sequences from those used in the primary screen.

### *In silico* protein-protein interaction and pathway analysis

Ingenuity Pathway Analysis (IPA, QIAGEN, RRID:SCR_008653) and STRING (RRID:SCR_005223) [23] analyses were performed to identify enriched pathways and protein–protein interactions among these 42 candidates. For IPA, a core analysis was performed using only direct relationships and used the approximately 21,584 genes represented in the screen as background. For STRING, relationships were mined using only experiment- and database-determined relationships of at least “medium confidence.”

### Cell viability and apoptosis assays

Cell viability was assessed with the CellTiter 96^®^ AQueous One Solution Cell Proliferation (Promega, Cat# G3582) following manufacturer’s recommendations. Briefly, cells were seeded into 96-well plates at 3,000 cells/well in complete medium and incubated for 24 hr. Each drug was then added at various concentrations to triplicate wells in a final volume of 100 μL of medium. After 72 hr of incubation, 20 μL of MTS tetrazolium reagent was added, and the incubation was continued for an additional 2 hr at 37°C. The absorbance of soluble MTS tetrazolium formazan produced by viable cells following drug exposure was measured at 490 nm using a microplate reader. The sensitivity of cells to each drug was expressed as a percentage of the vehicle-treated control. For apoptosis assays, after the indicated treatment, both floating and non-floating cells were collected and washed twice in PBS. The level of apoptosis was measured by flow cytometry using the Annexin V/PI assay (BD, Biosciences, Cat#556547) according to the manufacturer’s protocol using BD LSRII (BD, Biosciences) equipment. Data were collected on 10,000 cells and analyzed with FlowJo software (FlowJo, RRID:SCR_008520).

### Clonogenic survival assay

Cell lines were treated with drug under the conditions described during 24 hr, then cells were harvested in trypsin, counted, and reseeded in triplicate in 6-well plates at a density of 500 cells/well. Following 14 days of growth, surviving cells were stained with 1% crystal violet (Sigma-Aldrich, Cat# C0775) for 30 min, washed with distilled water, and air dried, Colonies containing more than 50 cells were scored and the total number of colonies per dish was normalized to untreated controls. Colony formation was quantified by analysis of scan images using Fuji Image J software (RRID:SCR_003070).

### Gene-specific RNAi and transfection

ATR siRNA transfections were performed as described for screening. The target sequences for the ATR siRNAs are indicated in Supplementary Dataset 2. Cells transfected with Ambion Silencer Select Negative Control #2 were used for comparison. After 48 hr the medium was removed, fresh medium containing etoposide (10 uM) or vehicle (0.1% DMSO) was added, and the cells were incubated at 37°C for additional 48 hr.

### Gene expression by qPCR

Total RNA was extracted from cells using the RNeasy Mini Plus Kit (QIAGEN, Cat#74034). Complementary DNAs were generated from 1 μg of purified RNA using iScript cDNA synthesis kit (BioRad, Cat# 1708890) following manufacturer’s recommendations. qPCR was performed on an HT7900 system (Applied Biosystems) using pre-validated primers for ATR (Hs00992123_m1) All reactions were done in triplicate, and relative quantification values were calculated based on the 2^−ΔΔCt^ method using expression from the housekeeping genes β-2-microglobulin (B2M, Hs00187842_m1) for normalization as previously described [54].

### Immunoblot analysis

Whole-cell lysates were prepared by lysing cells in RIPA Lysis and Extraction Buffer (Thermo Fisher, Cat# 89900) supplemented with 1× Halt Protease Inhibitor Cocktail (Thermo Fisher, Cat#87786) was used to determine protein concentrations. Equal protein amounts (15 μg) were separated on NuPAGE, Bis-Tris precast (4–12%) gels (Cat# NP0336PK2), transferred to polyvinylidene difluoride membranes (Cat#IB401032) using an iBlot Dry Blotting System (all from Thermo Fisher Scientific) and incubated with primary antibodies against the following proteins: p53 (DO-1, Santa Cruz Biotechnology Cat# sc-55476, RRID:AB_630061), actin (C-11, Santa Cruz Biotechnology Cat# sc-1615, RRID:AB_630835). Horseradish peroxidase linked goat anti-mouse (Santa Cruz Biotechnology Cat# sc-2005, RRID:AB_631736), and donkey anti-goat (Santa Cruz Biotechnology Cat# sc-2020, RRID:AB_631728) were used as secondary antibodies. Protein was detected using SuperSignal Chemiluminescent Substrate (Thermo Fisher, Cat# 34577).

### Statistical analysis

Analysis and estimation of the IC50 for single-agent analysis and drug combinations was performed using GraphPad Prism statistical software (GraphPad Prism, RRID:SCR_002798). Data are represented as mean ± SDs from at least three independent experiments unless otherwise indicated. One-way ANOVA with Tukey post hoc test was used for comparisons between groups.

## SUPPLEMENTARY MATERIALS







## Abbreviations

ATR: Ataxia-telangiectasia and the Rad3-related kinase
DDR: DNA damage response
DSB: DNA double strand breaks
ICE: *In vivo* Complex of Enzyme
MAD: median absolute deviation
SEL: Synthetic Enhanced Lethal siRNA: small interfering RNA
Top2: DNA topoisomerase II
Top2cc: Top2 covalent complexes
