# Supplementary material for: Etoposide-induced DNA damage is increased in p53 mutants: identification of ATR and other genes that influence effects of p53 mutations on Top2-induced cytotoxicity
Source: Oncotarget. 2022 Feb 14;13:332–46. doi: 10.18632/oncotarget.28195 (PMC8845119; doi:10.18632/oncotarget.28195)
Supplement: Supplementary file 1 [file oncotarget-13-28195-s001.pdf]

Etoposide-induced DNA damage is increased in p53 mutants: identification of ATR and other genes that influence effects of p53 mutations on Top2-induced cytotoxicity

SUPPLEMENTARY MATERIALS

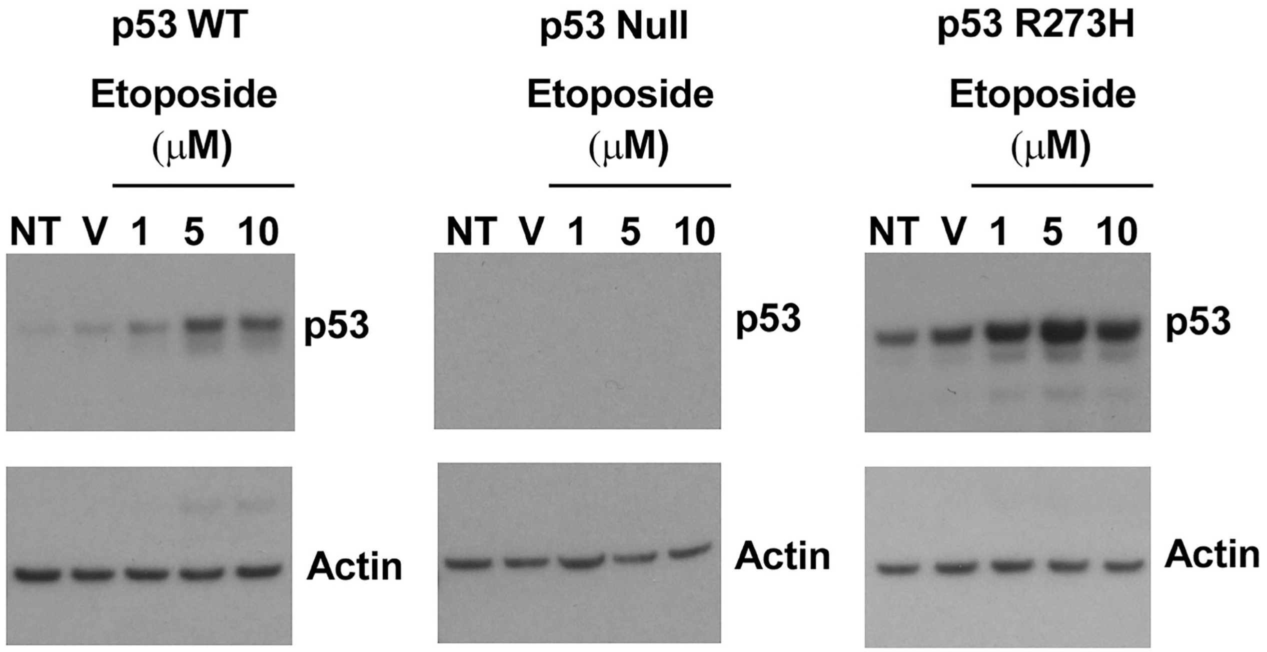

Supplementary Figure 1: p53 protein levels in isogenic HCT116 p53 WT, null and R273H cell lines treated with increasing doses of etoposide for 24 hr. Actin protein levels were used a loading control. Abbreviations: NT: no treatment; V: vehicle (DMSO). A representative image is shown.

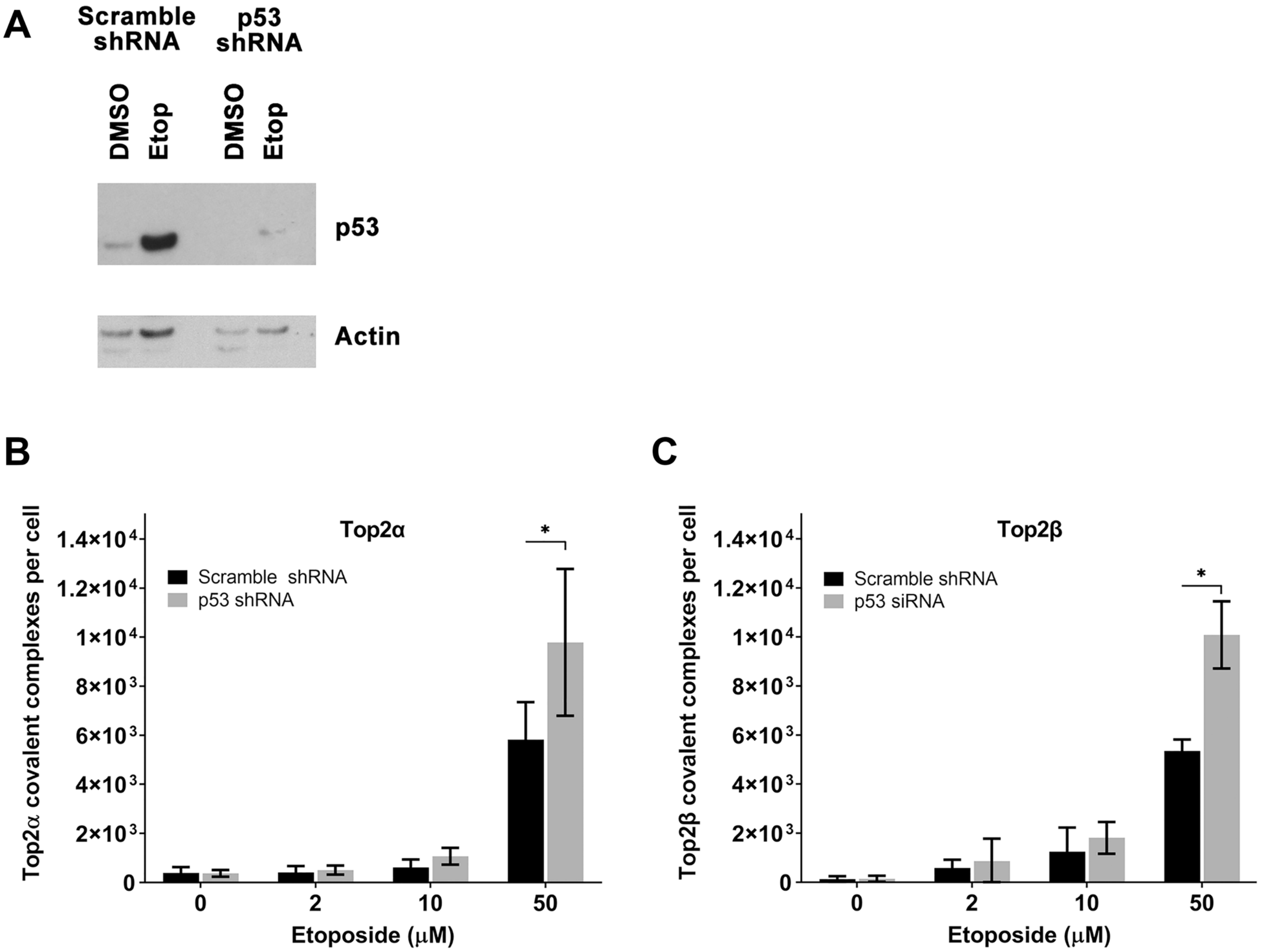

Supplementary Figure 2: Absense of functional p53 increases etoposide-induced Top2-DNA complexes formation in A549 cell lines. (**A**) p53 protein levels in A549 cells stably expressing scramble control shRNA or p53 proficient and p53 shRNA in response to etoposide (10 μM, 24 hr). The ICE assay was performed using isogenic A549 p53 proficient and p53 reduced cell lines following etoposide treatment (1 hr). ICE samples were isolated and levels of trapped Top2 isoforms were determined. ICE samples were isolated and levels of trapped (**B**) Top2α and (**C**) Top2β were determined. Data are presented as mean ± SDs from three separate experiments. *P* values < 0.05 were considered significant (*).

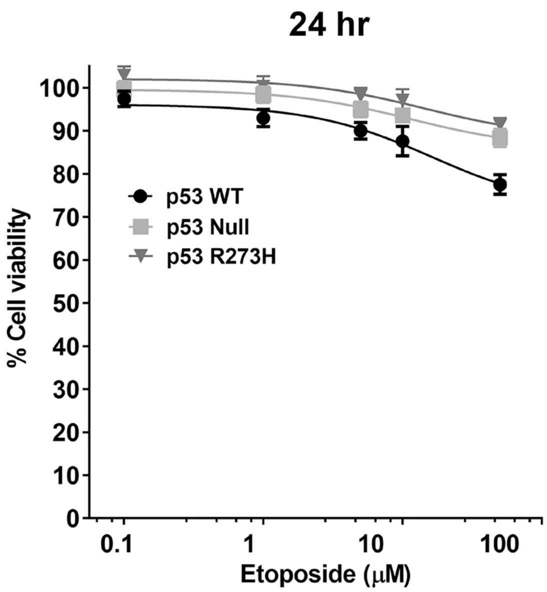

Supplementary Figure 3: TP53 genotype-dependent cytotoxic effect of etoposide in HCT116 colon cancer cells. Cell viablity and apoptosis were evaluated by MTS assay after 24 and 48 hr of incubation with etoposide at the doses (μM) indicated. Data are presented as mean + SDs of three separate experiments, carried out in triplicate. *P* values <0.001 were considered significant (*).

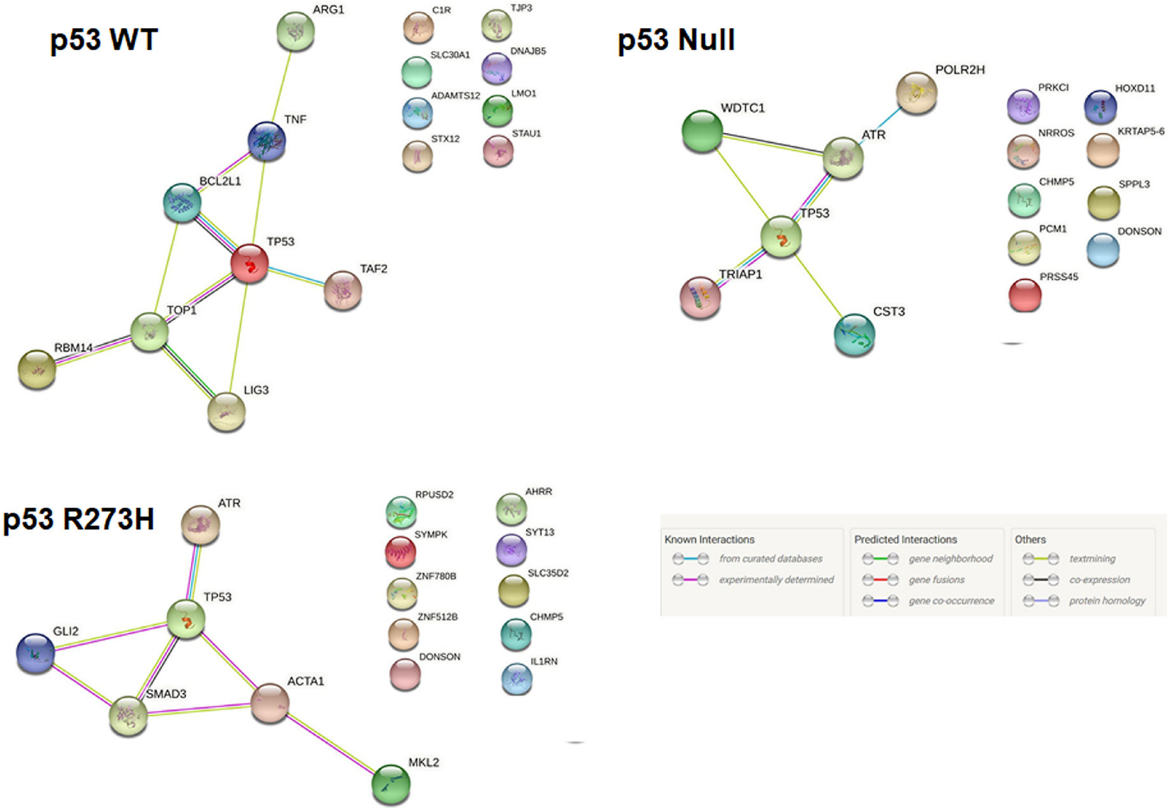

Supplementary Figure 4: Protein-protein network interaction analysis showing the inferred functional interaction (based on STRING) among SEL genes interacting with etoposide treatment. The top 15 potential etoposide SEL candidates for each HCT116 were inculded in the analysis using medium confidence STRING interactions (STRING integrated score >0.4).

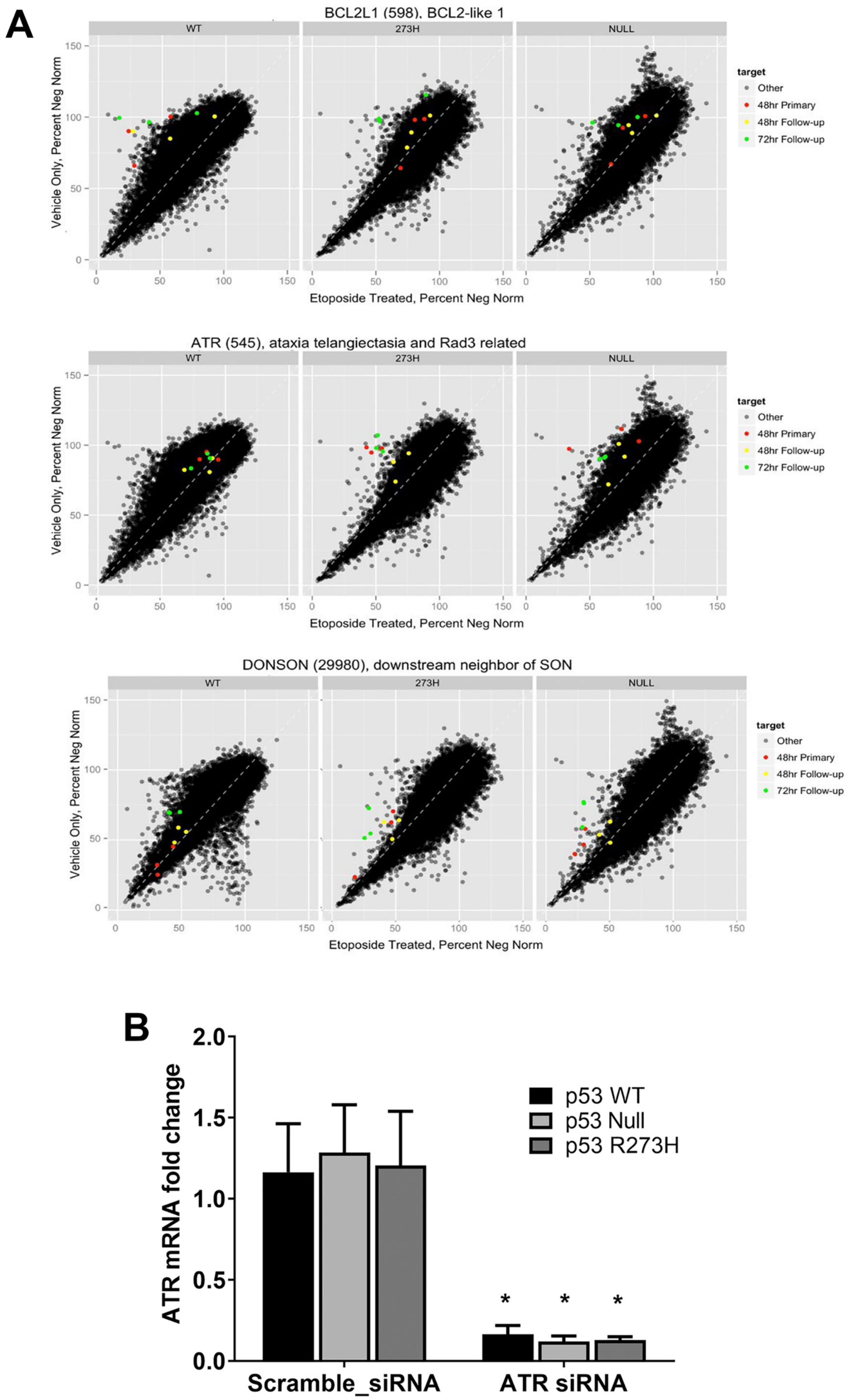

Supplementary Figure 5: Validation of ATR as an etoposide SEL target in p53 nonfunctional cells. (**A**) Representative plots of potential etoposide SEL targets. (**B**) Validation of ATR knockdown in isogenic HCT116 cell lines. Cells were transfected with a pool of siRNAs against ATR; after 72 hr ATR expression was evaluated by RT-PCR. Data are presented as mean ± SDs of three separate experiments, carried out in triplicate. *P* values <0.001 were considered significant when scramble siRNA vs. ATR siRNA was compared for each cell line (*).

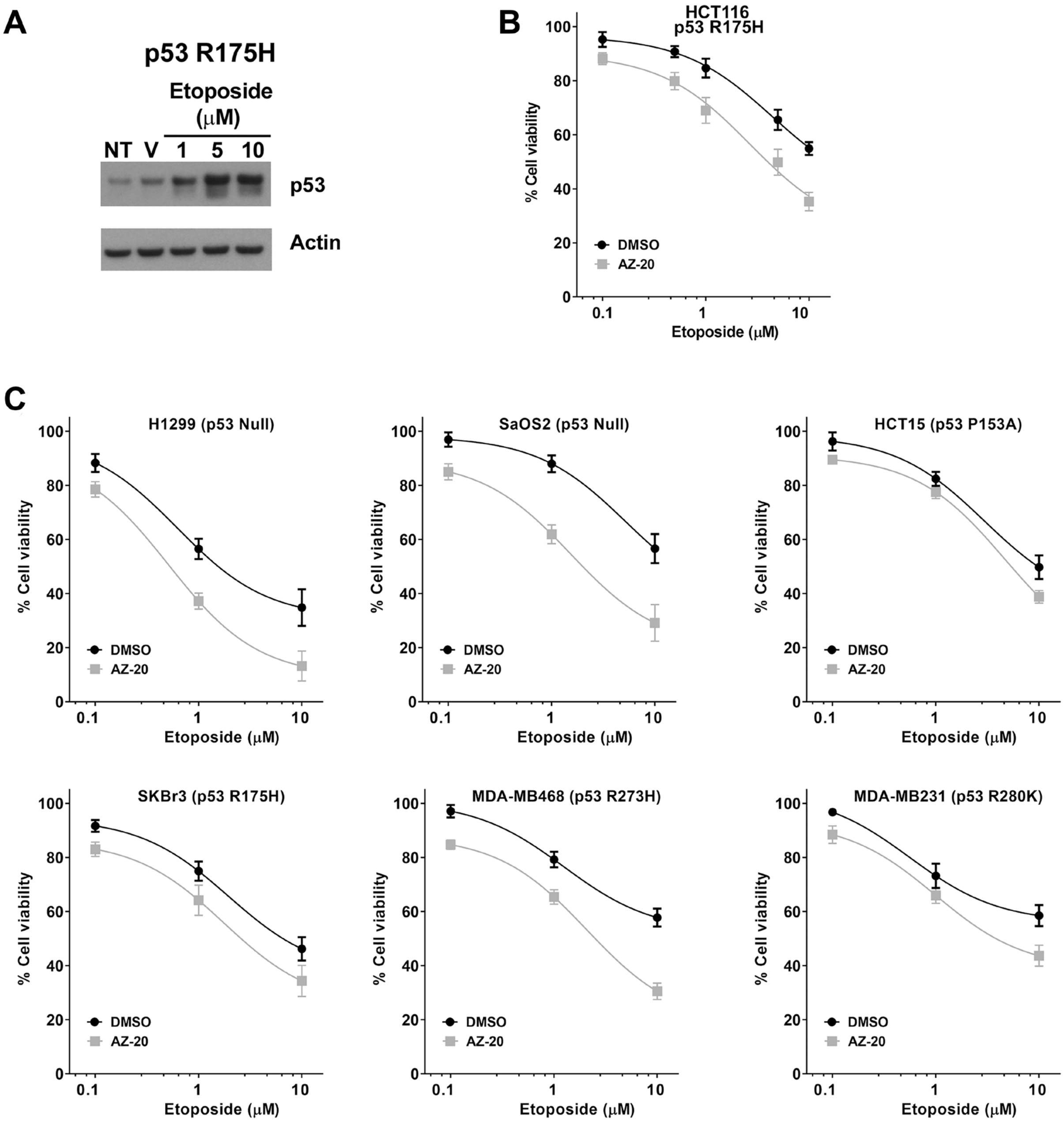

Supplementary Figure 6: Pharmacological inhibition of ATR increased etoposide sensitivity in human cancer cell lines with different p53 functional backgrounds. (**A**) p53 protein levels in HCT116 cells stably expressing R175H mutant p53 treated with increasing doses of etoposide for 24 hr. Actin protein levels were used as a loading control. Abbreviations: NT: no treatment; V: vehicle (DMSO). A representative image is shown. Cell viability curves were assessed by MTS assay following pretreatment with either DMSO or ATR inhibitor AZ-20 (5 nM) for 3h, followed by etoposide treatment (10 μM, 72 h) in (**B**) HCT116 cells stably expressing R175H p53 mutant and in (**C**) human cancer cell lines with different p53 mutations. Presented are the man ± SDs of three independent experiments carried out in triplicate.

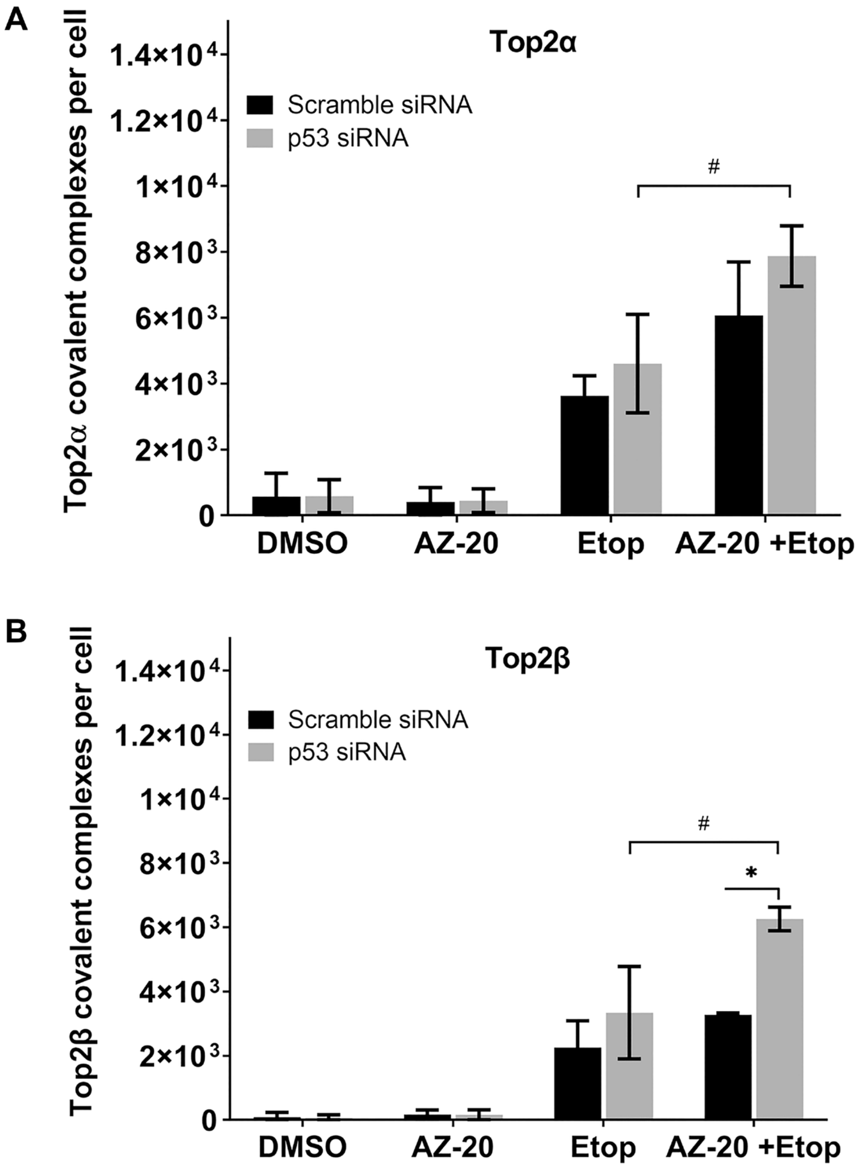

Supplementary Figure 7: Pharamacological inhibition of ATR increases levels of Top2cc in absence of functional p53. The ICE assay was performed using isogenic A549 cell lines following pretreatment with either DMSO or ATR inhibitor AZ-20 (200 μM) for 0.5 hr, followed by etoposide treatment (10 μM, 2 hr). ICE samples were isolated and levels of trapped (**A**) Top2α and (**B**) Top2β isoforms were determined. Data are presented as mean ± SDs from three separate experiments. *P* values <0.05 were considered significant (*,#).

Supplementary Dataset 1: Primary screen SEL etoposide p53 Menendez. See Supplementary Dataset 1

Supplementary Dataset 2: Validation phase SEL etoposide p53 Menendez. See Supplementary Dataset 2
